# Effect of RVAD Cannulation Length on Right Ventricular Thrombosis Risk: An In Silico Investigation

**DOI:** 10.1007/s10439-024-03474-4

**Published:** 2024-02-28

**Authors:** Kar Ying Thum, Sam Liao, Michael Šeman, Mehrdad Khamooshi, Josie Carberry, David McGiffin, Shaun D. Gregory

**Affiliations:** 1 Cardiorespiratory Engineering and Technology Laboratory, Department of Mechanical and Aerospace Engineering, Monash University, Melbourne, VIC Australia; 2 Department of Cardiothoracic Surgery and Transplantation, Alfred Hospital and Monash University, Melbourne, VIC Australia; 3 School of Public Health and Preventative Medicine, Monash University, Melbourne, Australia; 4 Department of Cardiology, Alfred Hospital, Melbourne, VIC Australia

**Keywords:** Right heart failure, Computational fluid dynamics (CFD), Flow modelling, Right ventricle diaphragmatic implantation, BiVAD, One-way fluid–structure interaction (FSI)

## Abstract

Left ventricular assist devices (LVADs) have been used off-label as long-term support of the right heart due to the lack of a clinically approved durable right VAD (RVAD). Whilst various techniques to reduce RVAD inflow cannula protrusion have been described, the implication of the protrusion length on right heart blood flow and subsequent risk of thrombosis remains poorly understood. This study investigates the influence of RVAD diaphragmatic cannulation length on right ventricular thrombosis risk using a patient-specific right ventricle in silico model validated with particle image velocimetry. Four cannulation lengths (5, 10, 15 and 25 mm) were evaluated in a one-way fluid–structure interaction simulation with boundary conditions generated from a lumped parameter model, simulating a biventricular supported condition. Simulation results demonstrated that the 25-mm cannulation length exhibited a lower thrombosis risk compared to 5-, 10- and 15-mm cannulation lengths due to improved flow energy distribution (25.2%, 24.4% and 17.8% increased), reduced stagnation volume (72%, 68% and 49% reduction), better washout rate (13.0%, 11.6% and 9.1% faster) and lower blood residence time (6% reduction). In the simulated scenario, our findings suggest that a longer RVAD diaphragmatic cannulation length may be beneficial in lowering thrombosis risk; however, further clinical studies are warranted.

**Supplementary Information:**

The online version contains supplementary material available at 10.1007/s10439-024-03474-4.

## Introduction

With the rising prevalence of heart failure worldwide, durable ventricular assist device (VAD) implantation is an alternative to cardiac transplantation for patients with advanced heart failure due in part to the shortage of donor hearts [23]. Despite favourable patient outcomes, right ventricular failure remains a common complication following left VAD (LVAD) implantation, with up to 11% of LVAD recipients subsequently requiring additional support on the right heart (BiVAD support) [30]. For patients who require long-term support of the right heart, rotary LVADs have been used off-label as a right VAD (RVAD) due to the lack of a clinically approved durable RVAD device. This off-label LVAD use has become increasingly prevalent in recent years with multiple operative approaches proposed for RVAD implantation [6, 22, 30].

Surgical approaches to RVAD cannulation with the HeartMate 3 VAD typically involve the reduction of inflow cannula length using a series of felt spacers interposed between the right atrial or right ventricular wall and the sewing ring. This was not necessary with the HeartWare HVAD since the inflow cannula length could be adjusted using the sewing ring. The reduction of the inflow cannula length is crucial during RVAD implantation to prevent impingement of the cannula on the right atrial or ventricular septum which could lead to inflow obstruction. To date, various RVAD cannulation lengths (both HeartWare HVAD and HeartMate 3) have been reported in clinical studies including 10 mm [18, 33] and 15 mm [5, 11, 17, 19] and cannulation without any spacer added which was performed with an RV diaphragmatic approach [3]. However, the impact of cannulation length on right ventricular blood flow dynamics, and the potential for thrombosis, has yet to be elucidated.

Thrombosis is a well-established complication after VAD implantation and remains a particular concern in the off-label use of LVAD as durable right heart support. RVAD-associated thrombosis could lead to ingestion of thrombus into the pump or pulmonary embolism which are associated with increased mortality [6, 7]. The incidence of RVAD thrombosis varies widely in the literature, ranging from 0% to 75% over a follow-up period of 6 to 24 months [6]. This variability in RVAD thrombosis rate may be attributed to several factors, including, but not limited to, the type of VAD used, pump operating conditions, patient disease state and underlying cardiovascular diseases. However, it should also be noted that the current non-standardized, off-label RVAD implantation techniques may contribute to this high variability.

Unfavourable blood flow patterns induced by the VAD cannula protrusion can generate regions of blood stasis that predispose to thrombus formation within the cardiac chamber [2, 20, 26]. As the blood flow dynamics can be influenced by RVAD surgical techniques, such as inflow cannulation site, length and angle, it is important to understand how each of these may affect the blood flow dynamics within the right heart. Whilst there are studies available showing that an increased LVAD cannulation length may be beneficial in reducing thrombosis within the left ventricle [14, 28], it is currently unknown whether RVAD cannulation length plays an important role in reducing thrombosis risk within the RV. Thus, this study aimed to investigate the effect of RVAD cannulation length on the RV flow dynamics and its implication on the thrombosis risk using a patient-specific blood flow simulation model. Results generated from the simulation offer insight regarding appropriate RVAD cannulation length which may contribute to a reduced thrombosis risk.

## Methods

### Geometry Construction

A computed tomography scan of a de-identified patient with temporary biventricular support was obtained (approved by Human Research Ethics Committee of the Alfred Hospital, Australia (Project 70/19)) to reconstruct the three-dimensional model of the RV using Mimics 21.0 (Materialise NV., Leuven, Belgium) and MeshMixer (MeshMixer Autodesk, Inc., Mill Valley, California, U.S.). The tricuspid valve was simplified as a two-dimensional orifice using the dimensions obtained from the patient data [8]. The right atrium was simplified as a column extending 40 mm from the tricuspid orifice. The pulmonary valve was not modelled as the valve was closed in the simulated full RVAD-supported scenario.

**Fig. 1:**
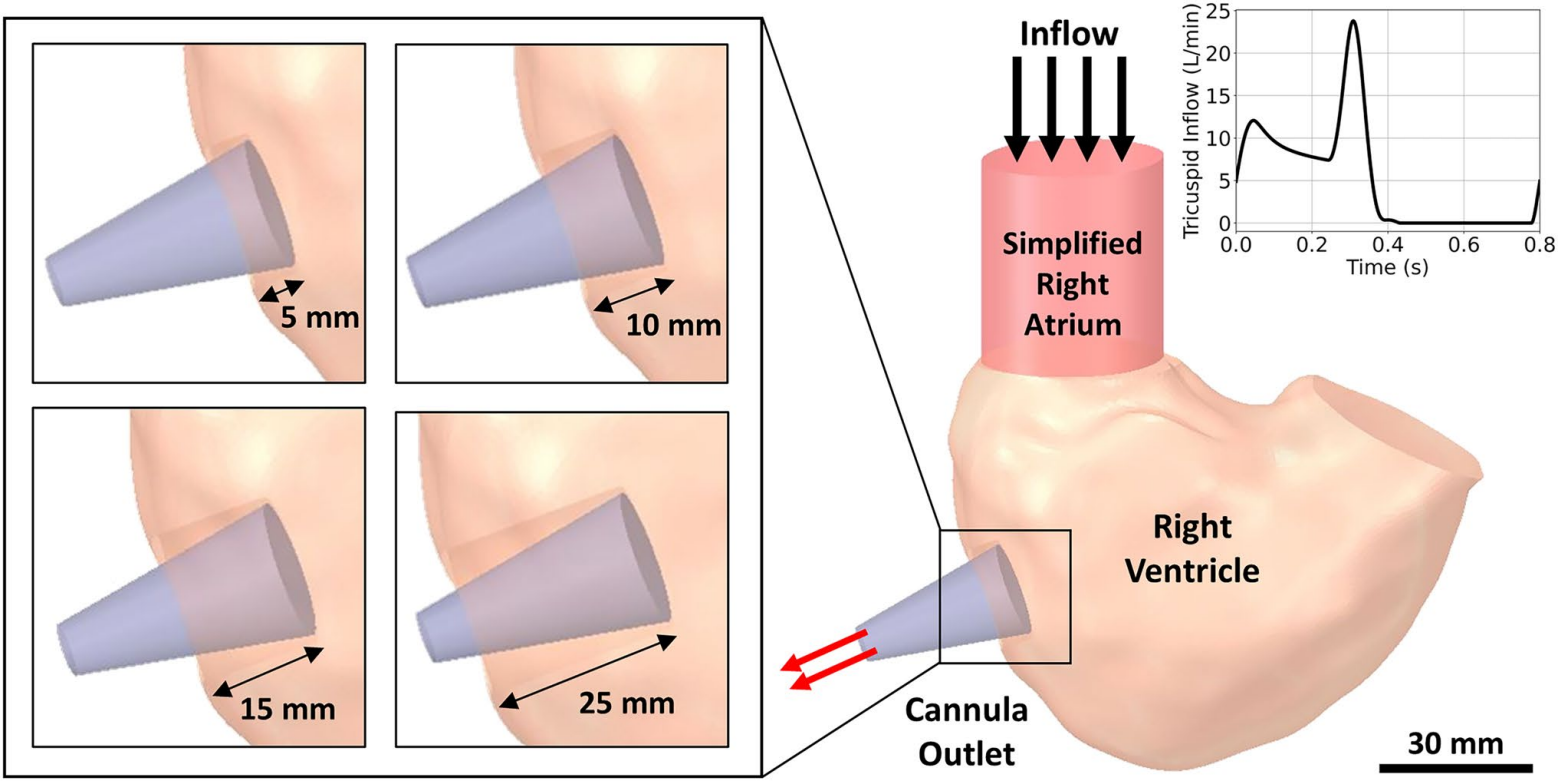
Virtual implantation of HeartMate 3 cannula in the right ventricle with a diaphragmatic approach. Internal fluid domain of the inflow cannula with different cannulation lengths are shown as grey on the left. The boundary condition for the tricuspid inflow is shown on the top right.

In this study, RV diaphragmatic cannulation site was chosen due to the lower suction events reported with the cannula lying in parallel to the ventricular septum [21], allowing maximum space for cannula insertion. A HeartMate 3 inflow cannula, featuring an outer diameter of 20.50 mm and an inner diameter of 18.50 mm that tapered down to an inner diameter of 7.30 mm over a length of 32 mm, was modelled. The cannula was then virtually implanted onto the diaphragmatic surface of the RV, angled towards the outflow tract. Four RVAD cannulation lengths were investigated – 5 mm, 10 mm, 15 mm and 25 mm, as shown in Fig. 1. Cannulation lengths were chosen based on expert opinion from an experienced cardiac surgeon and previous clinical studies [3, 17, 19, 33].

### Computational Model Setup

#### Boundary Conditions

To simulate a time-dependent physiological flow condition, a previously developed 0-dimensional closed-loop lumped parameter model (LPM) [16] was used to generate the hemodynamics of a HeartMate 3 biventricular assist device (BiVAD)-supported patient. In the adopted LPM, a time-varying elastance function was used to describe the contraction of the heart chambers. A non-linear end-diastolic pressure–volume relationship was defined for all the chambers, whilst a linear and curvilinear end-systolic pressure–volume relationship was defined for the atriums and ventricles, respectively [16]. The arteries and veins in the systemic and pulmonary compartments were described in terms of pressure, volumetric flow rate, vascular resistance, and compliance, with the fluid inertance only included in the aorta and pulmonary artery in which the blood acceleration was significant [16]. The tricuspid and pulmonary valves were modelled as object-oriented hydraulic valves using Simscape Fluids (MathWorks Inc., Massachusetts, U.S.), in which transvalvular flow is based on the pressure difference across the valve, accounting for flow dynamics, mass, and energy conservation [29]. The LPM parameter values used for simulating the condition are shown in the supplementary information Table S1 and S2. In brief, the end-systolic elastance (contraction force) of the left and right ventricles were reduced from a healthy baseline value (adopted from Lim et al. [16]) of 3.54 to 0.86 mmHg.ml^-1^ for the LV and 1.75 to 0.5 mmHg.ml^-1^ for the RV, exhibiting systolic dysfunction in both ventricles. Additionally, the unstressed end-systolic and diastolic volumes of the left and right ventricles were increased to replicate dilated ventricles of the patient with severe biventricular failure. To model a HeartMate 3 pump, a pump equation was extracted from the HQ curves of HeartMate 3 pump [34], describing the performance of the pump in terms of pump outflow rate, pressure head, and the pump speed. Two HeartMate 3 pumps were then incorporated into the LPM, each supporting the failing left and right ventricles at a speed of 5400 RPM and 4300 RPM, respectively, to simulate a full BiVAD-supported condition (all flow passed from RV to the pulmonary artery through the pump, instead of the pulmonary valve). The LPM was then solved in SIMULINK (MathWorks Inc., Massachusetts, U.S.). The simulated hemodynamics of a BiVAD-supported condition was comparable with the reported clinical data of BiVAD-supported patients [31], as shown in Table 1. The time-dependent tricuspid inflow and the RV volume waveform generated from the LPM were then implemented in the computational fluid dynamics (CFD) simulation as boundary conditions.

**Table 1:** Hemodynamic comparison of the simulated BiVAD-supported condition and the clinical data of BiVAD patients (HeartWare HVAD) [31].

Parameters	Simulated biventricular failure + BiVAD	Clinical data from Shehab et al., 2017 [31] (mean with standard deviation)
Heart rate (bpm)	75	76 (14)
Cardiac output (L/min)	5.09	5.6 (1.2)
Mean arterial pressure (mmHg)	86.6	77 (16)
Mean pulmonary arterial pressure (mmHg)	23.6	22 (7)
Left atrial pressure (mmHg)	14.5	13 (6)
Right atrial pressure (mmHg)	7.4	12 (5)
Systemic vascular resistance (mmHg.s.mL^-1^)	0.935	0.745 (0.257)
Pulmonary vascular resistance (mmHg.s.mL^-1^)	0.107	0.094 (0.040)

#### Meshing

**Fig. 2:**
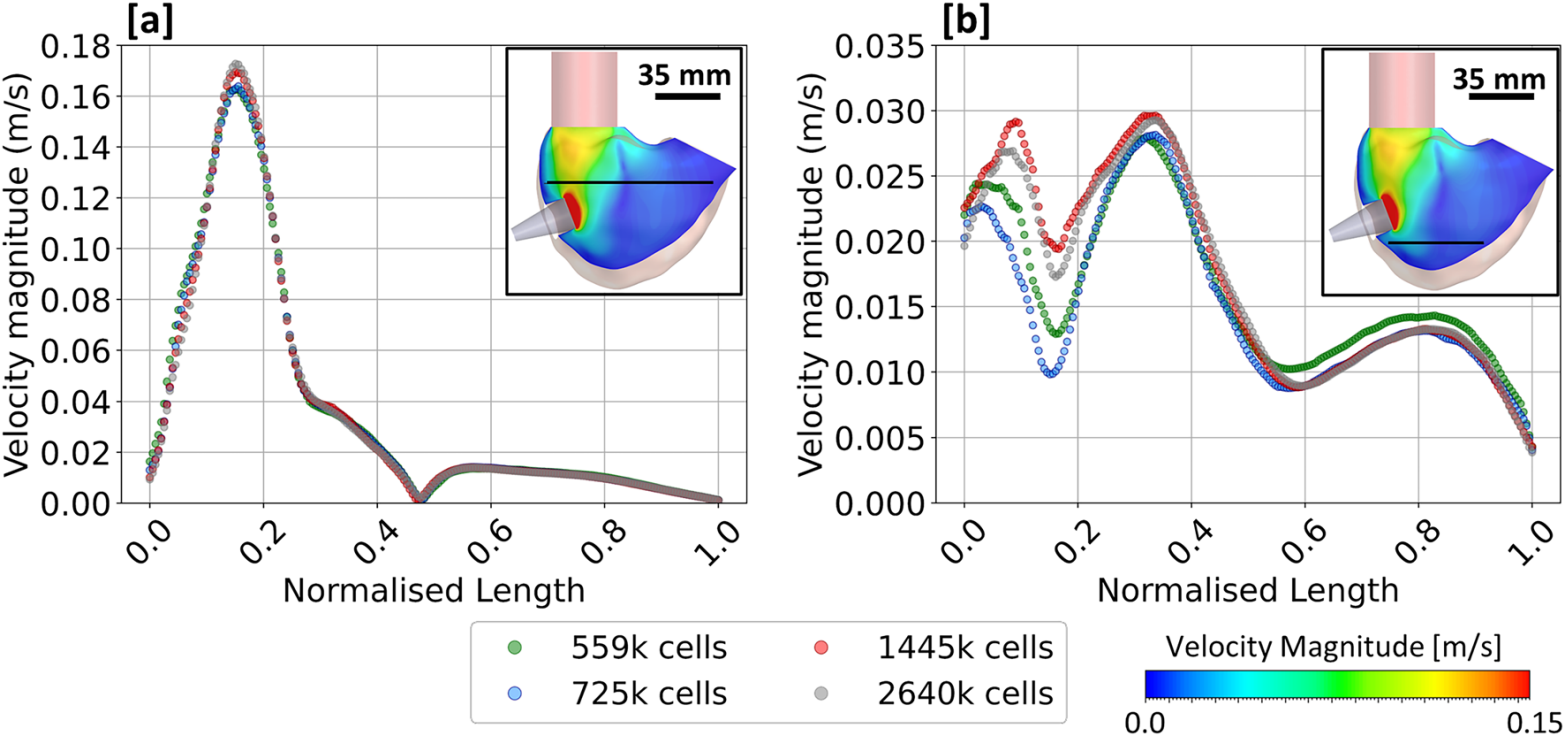
Mesh sensitivity study performed on the 10-mm cannulation length model. Velocity magnitude at **a** 30 mm—middle of RV and **b** 60 mm—near RV apex, below the tricuspid annulus, were compared on all the mesh sizes.

The geometry was meshed with tetrahedral elements using ANSYS Fluent 2019R2 (ANSYS Inc., Canonsburg, Pennsylvania, U.S.). Five prism layers were applied on the wall of the geometry using a smooth transition method with a ratio of 0.272 and a growth rate of 1.2. A mesh sensitivity study was performed on a rigid-walled model with transient tricuspid inflow (generated from LPM) prescribed at the inlet. Four different mesh sizes were generated on the 10-mm cannulation length RV geometry, resulting in 0.559, 0.725, 1.445 and 2.640 million cells. Mean velocities across two monitoring lines at 30 mm (middle of RV) and 60 mm (near RV apex) below the tricuspid annulus were compared for all mesh sizes, as shown in Fig. 2. Additionally, blood stagnation volume was also compared for the mesh sensitivity study, yielding values of 17.53 ml for 0.559 million cells, 17.92 ml for 0.725 million cells, 17.35 ml for 1.445 million cells and 16.75 ml for 2.640 million cells. This resulted in percentage differences of 4.7%, 7% and 3.6% when comparing the meshes with 0.559, 0.725 and 1.445 million cells to the mesh with 2.640 million cells, respectively. Therefore, the mesh consisting of 1.445 million cells was deemed appropriate as the average percentage difference for velocity and blood stagnation volume was less than 5% between 1.445 and 2.640 million cells.

#### One-way Fluid–Structure Interaction Model Setup

One-way fluid–structure interaction (FSI) simulation was performed in which only the displacement solution from the structural deformation was interpolated to the fluid mesh at the FSI surface, hence changing the volume of the fluid domain. The detailed simulation setup for the structural analysis and fluid flow is described in the following section. A time step size of 0.0002 s was used in the coupling simulation. Each time step had a maximum of 5 coupling iterations with a maximum iteration of 30 per coupling iteration. Convergence for the coupling simulation was achieved when the root-mean-square convergence for data transfer fell below 0.01.

#### Finite Element Model Setup

A wall thickness of 2 mm was generated and meshed from the surface of the RV geometry using ANSYS Meshing 2019R2 (ANSYS Inc., Canonsburg, Pennsylvania, U.S.), resulting in a RV wall with approximately 30937 tetrahedral elements. As the biomechanical analysis of the solid domain was not a focus in the study, but to impose a wall movement on the RV surface, the material property assigned to the RV wall was based on the approach by Lassila et al. [13]. A fixed support boundary condition was applied on the annuli of the tricuspid valve, pulmonary valve and the cannula–RV wall interface. An arbitrary pressure waveform which resembled the RV volume waveform generated from the LPM was applied outwardly onto the shell, simulating a severe failing RV movement with an ejection fraction of approximately 13% (stroke volume of 37 ml as obtained in the LPM). It must be noted that the solid domain was solely created to impose a displacement boundary condition for the fluid domain.

#### Fluid Model Setup

In the fluid model, the time-dependent tricuspid inflow generated from the LPM was prescribed at the inlet. The tricuspid valve was kept open, whilst the pulmonary valve was kept closed throughout the simulation given that the afterload from the RVAD in the LPM was too high for the valve to open in the simulated full BiVAD-supported condition. Blood was treated as non-Newtonian fluid, having a constant density of 1060 kg/m^3^. A Carreau model was used to describe non-Newtonian fluid behaviour using the following equation:

$$\eta = {\eta }_{\infty }+\left({\eta }_{0}- {\eta }_{\infty }\right){[1+{(\dot{\gamma} \lambda )}^{2}]}^{\frac{N-1}{2}},$$

where $\eta$ is the local viscosity (kg/ms), $\dot{\gamma}$ is the local shear rate (s^-1^), ${\eta }_{\infty }$ = 0.00345 kg/ms, ${\eta }_{0}$ = 0.056 kg/ms, $\lambda$ = 3.313 s and N = 0.3568 [4]. As the Reynolds number was higher than 4000 at the peak inflow, the kω Shear Stress Transport model was used for turbulence modelling. A second-order upwind scheme was defined for the spatial discretisation for pressure and momentum, whilst a second-order implicit scheme was defined for temporal discretisation. The simulation was solved using Pressure Implicit with Splitting of Operators (PISO) algorithm. The fluid model was first initialised for 8 s with a time step size of 0.0002 s, using a rigid wall model and a transient tricuspid inflow rate imposed at the inlet. Such initialisation was required to provide a stabilised solution for the subsequent one-way FSI simulation. Convergence was achieved when the residuals for the continuity, x, y, z velocity, k and omega fell below 10^-4^ in each time step. Thrombosis risk in each cannulation length was predicted and compared by analysing the velocity flow field, blood stagnation volume (defined as the time-averaged velocity of < 0.001 m/s and strain rate of < 100 s^-1^), time-averaged wall shear stress (TAWSS) at the cannula surface, washout rate and blood residence time [9, 14, 35].

### CFD Validation

A two-dimensional particle image velocimetry (PIV) experiment was performed to validate the simulation results produced by the CFD. Due to the complicated shape and movement of the RV, the validation was conducted on a rigid-walled cannulated RV model. The fabrication process for the rig was described in a previous study [35]. In brief, a 3D-printed RV model was placed into a clear acrylic box filled with clear silicone rubber (Solaris, Smooth-On, Inc., Macungie, Pennsylvania, U.S.) (Refractive Index = 1.41) and subsequently dissolved, leaving a negative mould of the RV.

**Fig. 3:**
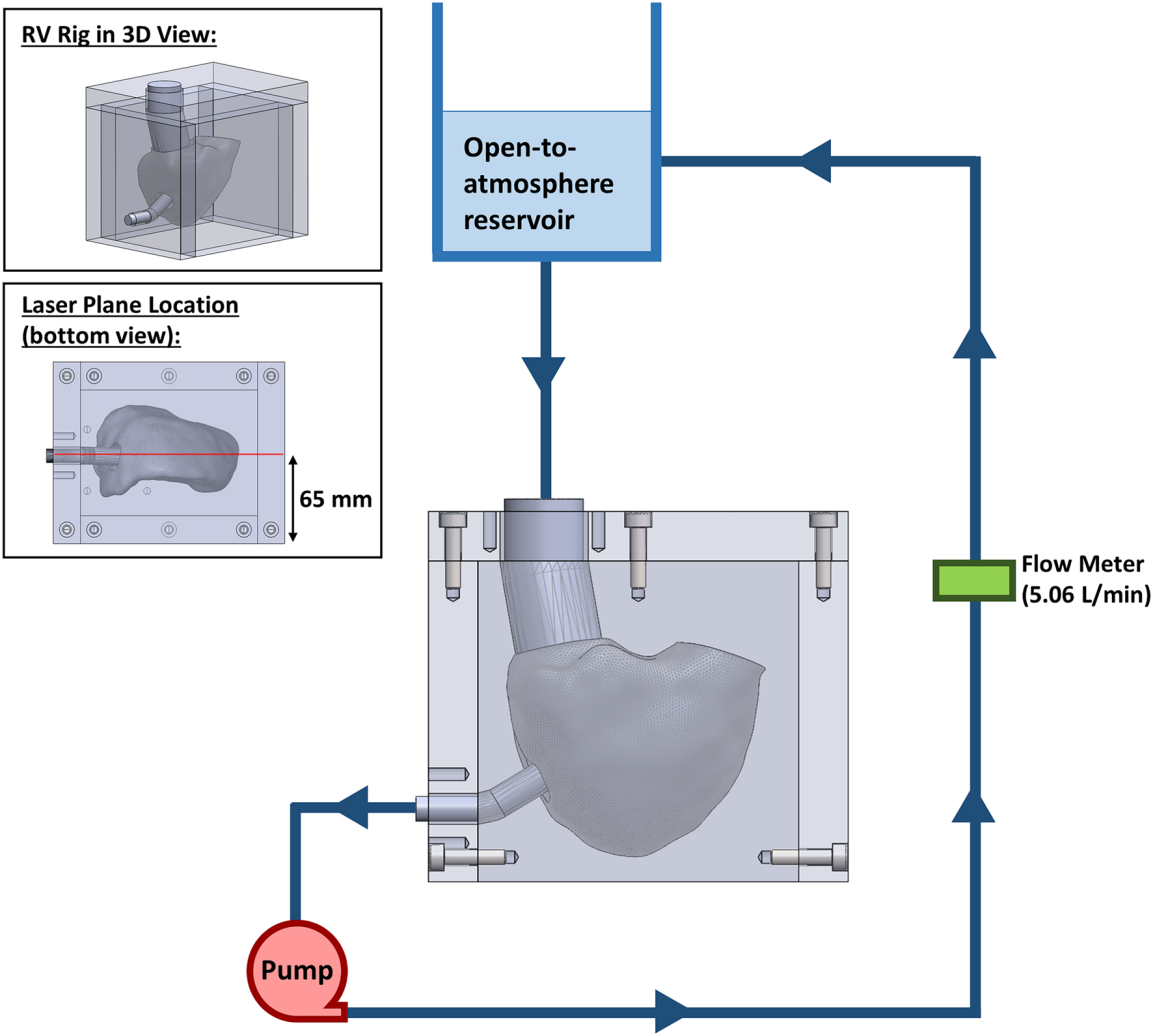
Schematic of the circulatory loop connected to the negative mould of the right ventricle.

A continuous flow circulatory loop was connected to the mould (Fig. 3), with a constant flow rate of 5.06 ± 0.05 L/min passing into the model. The loop was filled with a fluid mixture comprised 13.3%wt ammonium thiocyanate, 33.9%wt glycerol and 52.8%wt water, having a viscosity of 0.00277 Pa.s (DV2T, Brookfield Ametek, USA) and a refractive index of 1.4111 (Palm Abbe PA202, Misco, USA). 10-µm silver-coated hollow glass spheres (Dantec Dynamics, S-HGS-10, Denmark) were seeded into the fluid. A laser sheet generated by a double cavity 532-nm pulsed Nd:YAG laser (SpitLight Compact 200 PIV, Innolas, Germany) was passed through the centre of the RV model (laser plane as shown in Fig. 3), illuminating the particle flow behaviour in that plane of interest. 500 images pairs were captured at a frequency of 15 Hz, with a time delay of 1400 µs. An adaptive PIV algorithm (DynamicStudio 7.0, Dantec Dynamics, Denmark) was used to calculate the velocity vector map from each image pair, followed by statistical analysis to compute the mean velocity field. A CFD simulation was performed on the same RV geometry with flow rate and viscosity identical to the PIV setup. In-plane mean velocity contours and vectors across the middle plane of RV geometry were compared between the PIV and CFD results.

## Results

### Experimental Validation

**Fig. 4:**
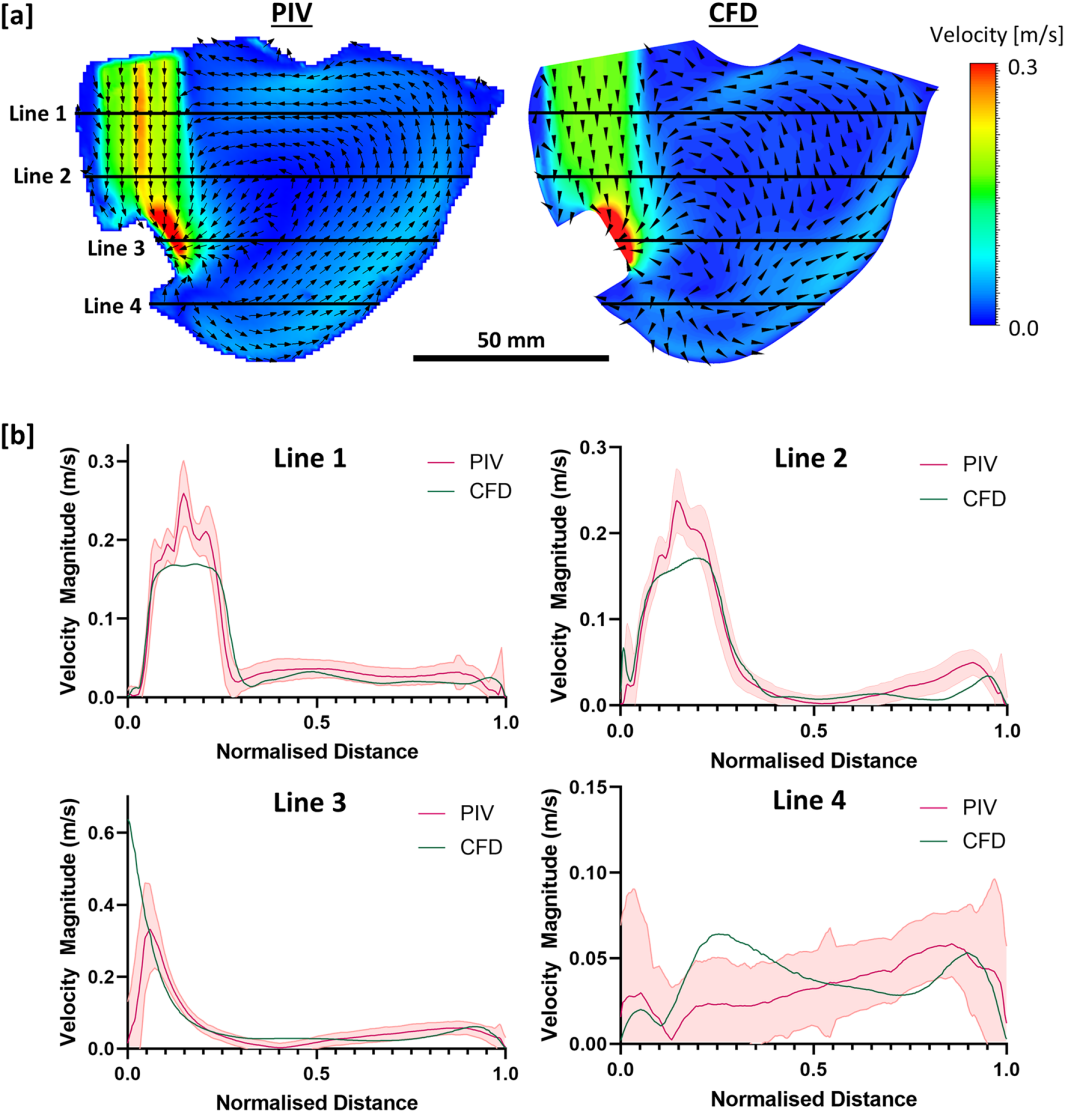
**a** Mean velocity contours and vectors through the centre of right ventricle from the experimental and simulation results. **b** Comparison of the velocity magnitude (with standard deviation) at different lines (approximately 14 mm, 30 mm, 46 mm and 63 mm below the tricuspid annulus).

The general flow patterns and mean velocity simulated in the CFD were comparable with the PIV results, as shown in Fig. 4. The velocity magnitude at lines 14 mm, 30 mm, 46 mm and 63 mm below the tricuspid annulus were also compared. Notably, similar inflow jet diameters were observed in both CFD and PIV, as illustrated in line 1 and line 2 in Fig. 4b. However, the maximum inflow jet velocity obtained in the CFD was slightly lower than the PIV results. Such discrepancies could be attributed to various sources of error, such as minor defects during the construction of the physical model or the calibration plane that did not perfectly coincide with the measurement plane, leading to an inaccurate scaling factor. A significant discrepancy in velocity at line 3 was also observed near the cannula inlet tip. A possible reason for this is that the seeding particles moved out of the interrogation area due to the much higher flow velocity at the tip, leading to a lower velocity being recorded in the PIV results compared to CFD. Despite the differences of velocity at certain points in the plane, the overall trend of PIV and CFD results are in good agreement, indicating that the CFD simulation has reasonable accuracy and validity in reproducing the physical flow behaviour.

### Velocity Fields

**Fig. 5:**
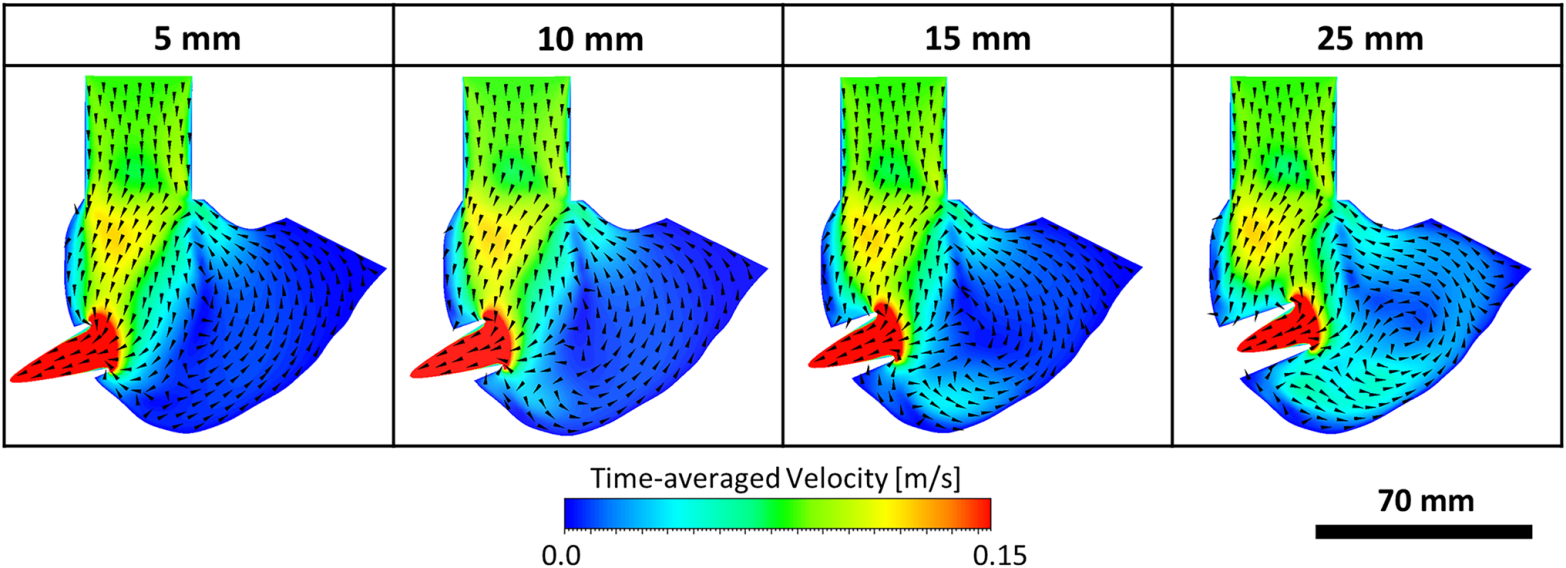
Comparison of the time-averaged flow velocity contour and vector field (over 5 cardiac cycles) in all cannulation length. Simulation results demonstrated that a longer cannulation length resulted in a higher flow velocity in the right ventricle apex and pulmonary valve region.

The general flow behaviour simulated within the RV in each cannulation length was illustrated by plotting a time-averaged velocity contour and vector field through the centre of the RV geometry, as shown in Fig. 5. It was observed that with a short cannulation length, most of the tricuspid inflow was immediately drawn out by the cannula without travelling further into the apex. In contrast, as the cannula was inserted deeper into the RV, some of the direct inflow appeared to avoid the suction from the cannula and travelled towards the apex and to the pulmonary valve region with higher flow velocities. The movement of blood within the RV was quantitatively evaluated by taking the volume integral of the flow kinetic energy (KE) within one cardiac cycle. The averaged KE (in a cardiac cycle) recorded with the 25-mm cannulation was 0.596 mJ, which was approximately 25%, 24% and 18% higher than 5-mm (0.476 mJ), 10-mm (0.479 mJ) and 15-mm (0.506 mJ) cannulation lengths, respectively. This showed that a longer cannulation length resulted in an overall higher flow energy distribution within the RV during a cardiac cycle.

### Vortex Formation

The formation of the three-dimensional vortex ring was visualised by plotting an iso-surface with a Q-criterion value of 325 s^-1^. Of note, the visualisation of vortices is not a metric to assess the thrombosis risk, but to visualize the recreation of vortex rings around the tricuspid annulus in the cardiac cycle. Although it is not a metric to assess thrombosis risk, it has been suggested in the literature that the formation of vortex plays an important role in enhancing blood mixing, which minimised thrombus formation [10, 24, 25]. In our simulation results, two vortex rings, taking the shape of the tricuspid annulus, were observed in all cannulation lengths during the cardiac cycle. The first vortex ring was formed around the tricuspid annulus after the E-wave peak as the inflow decelerated and rapidly dissipated as it travelled further into the RV. The second vortex ring formed after the A-wave peak in which the systolic phase commenced.

**Fig. 6:**
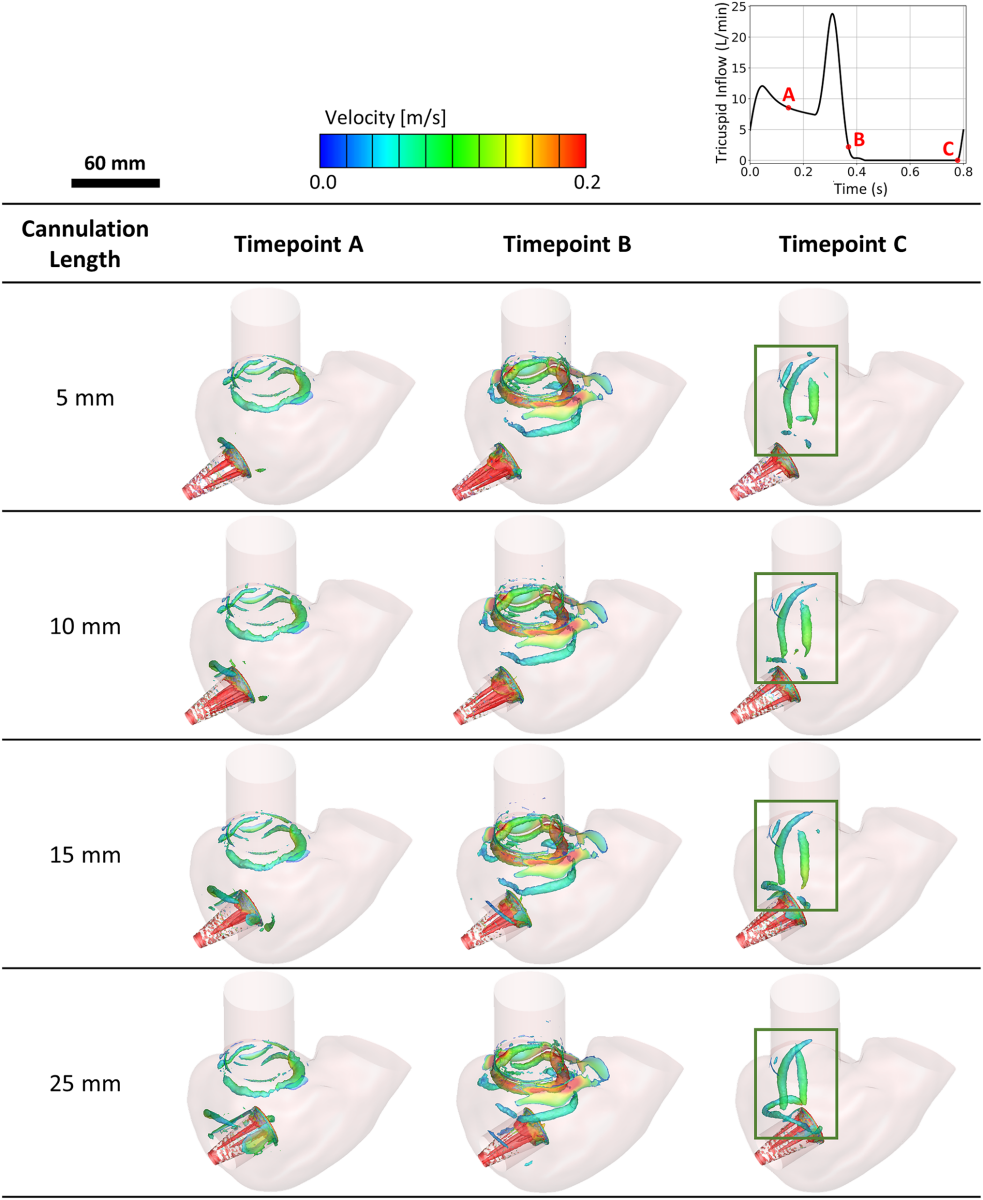
Vortex development at different timepoints (A, B and C) in a cardiac cycle, visualised with a Q-criterion value of 325 s^-1^. Tricuspid inflow for a single cardiac cycle, marked with timepoints A, B and C, is shown in top right. A diminished vortex structure was observed in shorter cannulation (particularly near the inflow cannula) as compared to a longer cannulation length.

In contrast to the first vortex ring, the diameter of the second vortex ring reduced and slowly collapsed into longitudinal vortex structures, stretching between the tricuspid annulus and the cannula, as the RV wall contracted. The longitudinal vortex structures were rapidly dissipated as the cardiac cycle approached the next E-wave peak. Although the behaviour of the vortex structures was similar in all cannulation lengths, a minor difference in the strength of the vortex structures was observed qualitatively; with a deeper cannulation length within the RV, the second vortex structures were comparatively larger than the short cannulation length (indicated within green box in Fig. 6—timepoint C).

### Stagnation Region and Time-averaged Wall Shear Stress

**Fig. 7:**
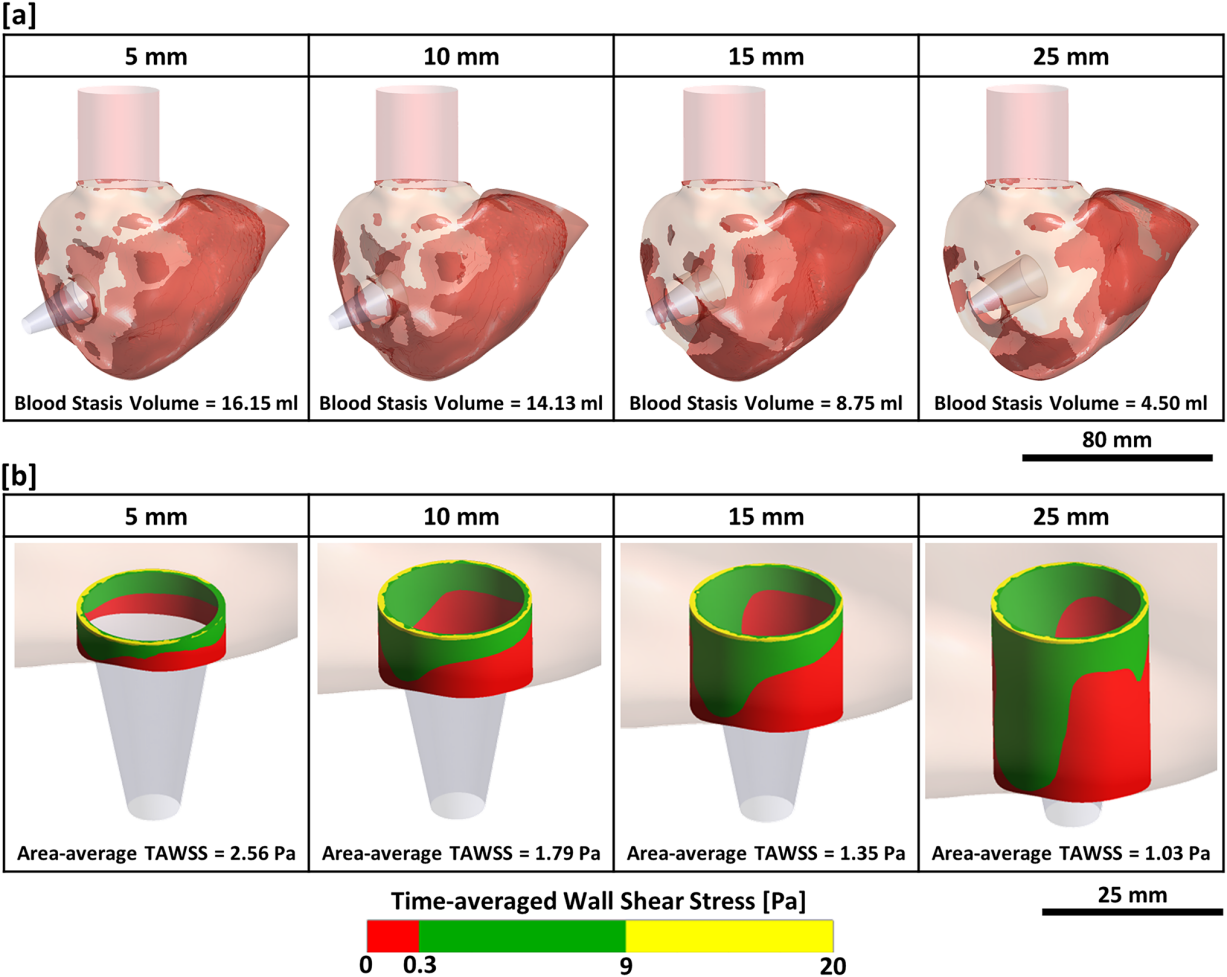
**a** Blood stagnation region (red) in all cannulation lengths with the total volume shown at the bottom. 25-mm cannulation length achieved the minimum stagnant blood volume as compared to other cannulation lengths. **b** TAWSS distribution on the cannula wall surface.

The potential region of blood stasis was predicted by identifying flow with a time-averaged velocity of less than 0.001 m/s and a strain rate of less than 100 s^-1^ [14]. It was observed that the volume of stagnant blood was reduced as the cannula was inserted deeper into the RV; this volume decreased from 16.15 to 4.50 ml (approximate 72% reduction) when the cannulation length was increased from 5 to 25 mm (Fig. 7a). With a longer cannulation length, the primary regions where the stagnant blood reduced was near the pulmonary valve and the RV apex. However, minor regions of stagnant blood were similarly found in all cannulation lengths, which were around the tricuspid annulus and the cannula–endocardium interface.

The area-weighted average TAWSS of the inflow cannula wall was computed and compared. A 59% of reduction in the TAWSS was observed as the cannulation length increased from 5 to 25 mm. The distribution of TAWSS on the inflow cannula wall surface was then classified into three different ranges: low non-physiological range (< 0.3 Pa), physiological range (0.3 < TAWSS < 9 Pa) and high non-physiological range (> 9 Pa), similar to the approach by Ghodrati et al. [9] (Fig. 7b). Regardless of cannulation lengths, it was observed that the tip of the inflow cannula experienced high non-physiological WSS, whilst the backside of the cannula (surface facing away from the tricuspid inflow) experienced low non-physiological WSS. However, it was found that an increase in cannulation length led to a larger exposure of the cannula surface area to low non-physiological WSS.

### Washout Rate

**Fig. 8:**
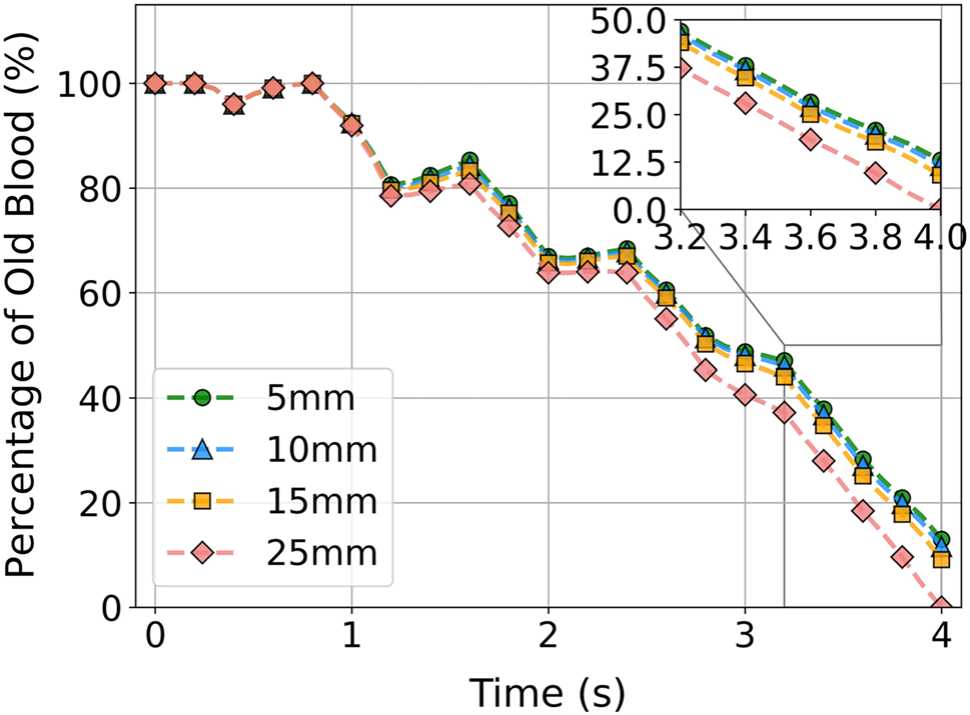
Comparison of the washout rate for all the cannulation lengths over 5 cardiac cycles (each cycle = 0.8 s).

The washout rate within the chambers was defined using the method described in previous studies [14, 35]. The trend of improved washout rate with increasing cannula insertion length is shown in Fig. 8. In the first cycle, there was no difference in the washout rate observed between all cannulation lengths; however, the washout effect became more evident with subsequent cycles. At the end of the simulation (after 5 cardiac cycles), approximately 13%, 12% and 9% of old blood still remained within RV for 5-mm, 10-mm and 15-mm cannulation length, respectively. However, all the old blood had been replaced by new blood in the 25-mm cannulation length.

### Blood Residence Time

Blood residence time was analysed using an Eulerian model, as described in previous studies [14, 35]. At the end of the simulation, a volume-weighted average blood residence time was computed to determine the duration blood spent in the domain. It was observed that the volume-averaged blood residence time within the RV for 5-, 10- and 15-mm cannulation lengths were similar, having values of 3.09 s, 3.08 s and 3.04 s, respectively. However, when the cannulation length was increased to 25 mm, the volume-averaged blood residence time reduced to 2.89 s, which is approximately 6% lower compared to the 5-mm cannulation length.

## Discussion

Thrombus formation within a VAD-supported chamber often complicates the treatment, particularly in patients receiving BiVAD support who are at a high risk of mortality [32]. It has been suggested that unfavourable blood flow dynamics within the heart chambers induced by the VAD cannula protrusion may generate regions of blood stasis that promote thrombosis [2]. With various RVAD surgical techniques being proposed, it remains uncertain whether the RVAD cannulation length could be a potential risk factors for thrombosis in a VAD-supported RV. In our simulation setting, a longer RVAD diaphragmatic cannulation length was demonstrated to improve the RV blood flow dynamics, reduce stagnant blood volume, improve the washout rate, and lower blood residence time within a patient-specific RV model. This suggests that the RVAD cannulation length can be enhanced to reduce thrombosis risk in RVAD implantation.

As reported in a previous clinical study, a longer LVAD inflow cannula in the LV demonstrated lower rates of thromboembolic adverse events and hence, better survival rates in LVAD patients [28]. Such clinical findings are consistent with the in silico results obtained by Liao et al. [14] in which a longer LVAD cannulation length demonstrated a lower thrombosis risk due to a higher LV washout and lower blood residence time. Interestingly, our simulation results revealed a similar finding in which a longer RVAD diaphragmatic cannulation length may be beneficial in lowering thrombosis risk within the RV, despite the anatomical difference between the LV and RV and the cannulation position. This observation might be attributed to the flow path taken between the tricuspid inflow and the cannula tip, where a longer path could be beneficial, as proposed by Liao et al. [14]. In cases of short RVAD diaphragmatic cannulation length, the resulting direct flow path from the tricuspid valve to the cannula tip was deemed undesirable based on our simulation. This configuration was of particular concern in patients with severe right ventricular failure with decreased ventricular wall motion as it could lead to inadequate ventricular washout and longer blood residence time, increasing the risk of thrombus formation within ventricle. Conversely, a longer diaphragmatic cannulation length may be more favourable as a longer flow path was achieved whilst maintaining a higher inflow momentum, improving ventricular washout and preventing blood stagnation around the RV apex (a region that is susceptible to blood pooling in dilated/restrictive cardiomyopathy). Additionally, the streamlined geometry of the RV may also facilitate an efficient redirection of inflow momentum from the apex towards the pulmonary valve, leading to a decrease in stagnant blood in the sub-valvular region and better ventricular washout. Whilst it has been suggested that vortex formation in the ventricle enhances blood mixing and thereby improving the ventricular washout [10, 25], the improved washout rate achieved with longer diaphragmatic cannulation length is likely attributed to the longer flow path instead of the vortex strength, given that the vortex structures were mainly confined between the tricuspid annulus and inflow cannula. Therefore, inducing a longer flow path by manipulating the cannulation length may be beneficial in mitigating thrombosis risk in an RVAD diaphragmatic cannulation.

In our simulated RVAD-supported condition, the RV apex, right ventricular outflow tract and the cannula–endocardium interface regions were predicted to be most susceptible to thrombus formation. Whilst the results suggested that increasing the diaphragmatic cannulation length could potentially lower blood stagnation around the apex and pulmonary valve region, it is anticipated that adjusting the RVAD support to allow intermittent opening of the pulmonary valve could also help to prevent sub-valvular blood stagnation which may be beneficial for patients with residual cardiac function [27]. Additionally, such intermittent opening of the valve has been suggested to improve the washout rate within the supported chamber [27]. For the thrombus formation around the cannula–endocardium interface (wedge thrombus), the inflow cannula design could be a contributing factor to an increased risk of wedge thrombus formation. Cannula surface facing away from the inflow are prone to flow recirculation, low WSS and stagnation. Increasing the cannulation length could elevate the risk of thrombus formation around the backside of cannula wall. The high non-physiological WSS experienced at the inflow cannula tip could increase the risk of platelet activation as the blood travels into the pump and potentially increase the risk of thrombus formation within the pump. Therefore, enhancing the cannula design, as demonstrated by Liao et al. through the use of an inferiorly flared inflow cannula and minimising the complexity of inlet tip, may potentially reduce the likelihood for thrombus formation around the cannula wall [15].

The balloon-like motion of the RV wall in the simulation model remains one of the limitations in this study; the septum wall moved without any countering pressure from the left ventricle. The outwards and elongation movement of the RV free wall is expected to be greater than the movement of the septum wall during diastole [12]. However, given that the condition simulated in the study was a patient with severe RV failure with an ejection fraction of 13%, it is anticipated that the results would have minimal effect. Secondly, it was suggested that the helical flow formation in the right atrium preserves flow kinetic energy and provides effective flow ejection into the RV [1]. Therefore, the simplification of the right atrium geometry in the simulation model may affect the subsequent flow direction, generating different flow fields in the RV. Limitations of this study also include the use of a single patient-specific geometry and the absence of the valve leaflets (tricuspid and pulmonary) geometry and movement; having an intermittent opening of the pulmonary valve could impact the overall flow development within the RV and potentially lead to different outcomes. As the study only focused on RV flow features that may contribute to thrombosis, the risk of wall suction, which is not within the scope of the study, should be taken into consideration when advancing the cannula further into the chamber, particularly in patients with a smaller size RV. Additionally, there are limitations present in the validation process. Firstly, the validation did not adhere to the international standard (ASME V&V40) for assessing numerical model credibility. Secondly, due to the challenges associated with conducting a PIV experiment on a compliant VAD-supported RV model which could introduce additional sources of error, we performed fundamental PIV validation using a rigid-walled RV model. However, this setup was different from the deformable CFD model used in the cannulation length investigation. Therefore, the simulation results should be interpreted with caution. An improved experimental setup that is more closely resembles the deformable CFD model is required for better validation of the results. Future work may also include investigations into different RV sizes, heart failure conditions and the inclusion of an anatomical dynamic tricuspid valve model.

In conclusion, this numerical study demonstrated that an improved flow distribution, reduced blood stagnation volume, reduced blood residence time and improved RV washout were achieved by inserting the cannula (RV diaphragmatic cannulation) deeper into the RV in a severe biventricular failure simulation. This study highlights the impact of inflow cannula length on RV flow dynamics and should be carefully considered during RVAD implantation. Further clinical studies investigating the impact of RV cannulation length on patient outcomes are warranted.

### Supplementary Information

Below is the link to the electronic supplementary material.

Supplementary file1 (DOCX 27 kb)

## Abbreviations

VAD: Ventricular assist device
RV: Right ventricle
LVAD: Left ventricular assist device
BiVAD: Biventricular assist device
RVAD: Right ventricular assist device
FSI: Fluid–structure interaction
LPM: Lumped parameter model
PISO: Pressure implicit with Splitting of Operators
PIV: Particle image velocimetry
KE: Kinetic energy
