# Supplementary material for: Effect of RVAD Cannulation Length on Right Ventricular Thrombosis Risk: An In Silico Investigation
Source: Ann Biomed Eng. 2024 Feb 28;52(6):1604–16. doi: 10.1007/s10439-024-03474-4 (PMC11082033; doi:10.1007/s10439-024-03474-4)
Supplement: Supplementary file 1 — Supplementary file1 (DOCX 27 kb) [file 10439_2024_3474_MOESM1_ESM.docx]

**Effect of RVAD Cannulation Length on Right Ventricular Thrombosis Risk: An In-silico Investigation**

**Kar Ying Thum^1^, Sam Liao^1^, Michael Šeman^1,3,4^, Mehrdad Khamooshi^1^, Josie Carberry^1^, David McGiffin^2^, Shaun D. Gregory^1^**

^1^ Cardiorespiratory engineering and technology laboratory, Department of Mechanical and Aerospace Engineering, Monash University, Melbourne, Victoria, Australia.

^2^ Department of Cardiothoracic Surgery and Transplantation, Alfred Hospital and Monash University, Melbourne, Victoria, Australia.

^3^ School of Public Health and Preventative Medicine, Monash University, Melbourne, Australia.

^4^ Department of Cardiology, Alfred Hospital, Melbourne, Victoria, Australia.

Email: [karying.thum@monash.edu](mailto:karying.thum@monash.edu)

# Supplementary Material

Table S1: Parameter values used in the LPM for simulating BiVAD supported conditions.

| **Parameter (unit)** | **Description** | **BiF + HM3** |
| --- | --- | --- |
| T_sys0_ (s) | Maximum systolic heart period | 0.55 |
| k_sys_ (s^2^) | Systolic heart period-inverse heart period slope | 0.075 |
| k_r,v_ | Proportion of ventricular systolic time to reach maximal contraction | 0.5 |
| k_r,a_ | Proportion of atrial systolic time to reach maximal contraction | 0.5 |
| P_0, pcd_ (mmHg) | Pericardial end-diastolic stiffness scaling term | 0.5 |
| V_usv_ (ml) | Systemic vein unstressed volume | 1995 |
| V_uao_ (ml) | Aortic unstressed volume | 201.75 |
| E_sv_ (mmHg.ml^-1^) | Systemic vein elastance | 0.013 |
| V_uvc_ (ml) | Vena cava unstressed volume | 136.17 |
| R_sv_ (mmHg.s.ml^-1^) | Systemic vein resistance | 0.095 |
| V_usa_ (ml) | Systemic arterial unstressed volume | 231.04 |
| V_upu_ (ml) | Pulmonary veins unstressed volume | 132.39 |
| V_upa_ (ml) | Pulmonary arterial unstressed volume | 91.67 |
| R_mt_ (mmHg.s.ml^-1^) | Mitral valve resistance | 0.01 |
| β_rvf_ (ml^-1^.mmHg^-1^) | RV end-systolic stiffness denominator coefficient | 0.000048 |
| R_n, ra_ (mmHg.s.ml^-1^) | Vena cava resistance | 0.012 |
| λ_lvf_ (ml^-1^) | LV end-diastolic stiffness coefficient | 0.038 |
| E_pu_ (mmHg.ml^-1^) | Pulmonary vein elastance | 0.04 |
| λ_rvf_ (ml^-1^) | RV end-diastolic stiffness coefficient | 0.032 |
| E_pa_ (mmHg.ml^-1^) | Pulmonary arterial elastance | 0.15 |
| E_es, ra_ (mmHg.ml^-1^) | RA end-systolic elastance | 0.20 |
| λ_la_ (ml^-1^) | LA end-diastolic stiffness coefficient | 0.045 |
| E_es, la_ (mmHg.ml^-1^) | LA end-systolic elastance | 0.20 |
| R_av_ (mmHg.s.ml^-1^) | Aortic valve resistance | 0.025 |
| V_0, ra_ (ml) | RA end-diastolic volume at zero pressure | 40 |
| R_pu_ (mmHg.s.ml^-1^) | Pulmonary vein resistance | 0.012 |
| P_0, la_ (mmHg) | LA end-diastolic stiffness scaling term | 0.50 |
| L_ao_ (mmHg.s^2^.ml^-1^) | Aortic inertance | 0.0022 |
| V_d, la_ (ml) | LA end-systolic volume at zero pressure | 25 |
| λ_ra_ (ml^-1^) | RA end-diastolic stiffness coefficient | 0.042 |
| P_0, ra_ (mmHg) | RA end-diastolic stiffness scaling term | 0.50 |
| V_0, pcd_ (ml) | Pericardial end-diastolic volume at zero pressure | 200 |
| V_0, la_ (ml) | LA end-diastolic volume at zero pressure | 35 |
| V_0, spt_ (ml) | Septal end-diastolic volume at zero pressure | 8 |
| V_d, spt_ (ml) | Septal end-systolic volume at zero pressure | 8 |
| V_d, ra_ (ml) | RA end-systolic volume at zero pressure | 35 |
| E_es, spt_ (mmHg.ml^-1^) | Septal end-systolic elastance | 19.5 |
| λ_pcd_ (ml^-1^) | Pericardial end-diastolic stiffness coefficient | 0.005 |
| L_pa_ (mmHg.s^2^.ml^-1^) | Pulmonary arterial inertance | 0.0018 |
| λ_spt_ (ml^-1^) | Septal end-diastolic stiffness coefficient | 0.17 |
| P_0, spt_ (mmHg) | Septal end-diastolic stiffness scaling term | 0.46 |
| V_total_ (ml) | Total blood volume | 5700 |
| α_lvf_ (mmHg^-1^) | LV end-systolic stiffness denominator term | 0.0040 |
| R_sa_ (mmHg.s.ml^-1^) | Systemic peripheral resistance | 0.63 |
| β_lvf_ (ml^-1^.mmHg^-1^) | LV end-systolic stiffness denominator coefficient | 2.2E-05 |
| R_n, pa_ (mmHg.s.ml^-1^) | Pulmonary peripheral resistance | 0.095 |
| V_0, lvf_ (ml) | LV end-diastolic volume at zero pressure | 145.0 |
| V_0, rvf_ (ml) | RV end-diastolic volume at zero pressure | 257.5 |
| E_sa_ (mmHg.ml^-1^) | Systemic arterial elastance | 0.37 |
| V_d, rvf_ (ml) | RV end-systolic volume at zero pressure | 215 |
| V_d, lvf_ (ml) | LV end-systolic volume at zero pressure | 120 |
| α_rvf_ (mmHg^-1^) | RV end-systolic stiffness denominator term | 0.008 |
| P_0, rvf_ (mmHg) | RV end-diastolic stiffness scaling term | 0.91 |
| R_ao_ (mmHg.s.ml^-1^) | Aortic resistance | 0.2 |
| P_0, lvf_ (mmHg) | LV end-diastolic stiffness scaling term | 0.98 |
| E_ao_ (mmHg.ml^-1^) | Aortic elastance | 0.8 |
| E_vc_ (mmHg.ml^-1^) | Vena cava elastances | 0.03 |
| E_es, lvf_ (mmHg.ml^-1^) | LV end-systolic elastance | 0.86 |
| E_es, rvf_ (mmHg.ml^-1^) | RV end-systolic elastance | 0.5 |

Table S2: Parameter values used in the Simscape fluids sub-section for modelling the tricuspid and pulmonary valves.

| **Parameter (unit)** | **Values** |
| --- | --- |
| Blood density (kg/m^3^) | 1060 |
| Kinematic viscosity (cP) | 3.5 |
| Tricuspid valve cracking pressure differential (psi) | 0.0001 |
| Tricuspid valve maximum opening pressure different (psi) | 0.15 |
| Tricuspid valve maximum opening area (cm^2^) | 5.0 |
| Tricuspid valve leakage area (cm^2^) | 1e-13 |
| Tricuspid valve discharge coefficient | 0.7 |
| Tricuspid valve critical Reynolds number | 4000 |
| Pulmonary valve cracking pressure differential (psi) | 0.01 |
| Pulmonary valve maximum opening pressure different (psi) | 0.25 |
| Pulmonary valve maximum opening area (cm^2^) | 4.0 |
| Pulmonary valve leakage area (cm^2^) | 1e-13 |
| Pulmonary valve discharge coefficient | 0.85 |
| Pulmonary valve critical Reynolds number | 4000 |

Table S3: Parameters used in mesh sensitivity study.

| **Model** | **Mesh 1** | **Mesh 2** | **Mesh 3** | **Mesh 4** |
| --- | --- | --- | --- | --- |
| Number of cells | 559288 | 724518 | 1444723 | 2640280 |
| Right ventricle body size (mm) | 2.00 | 1.75 | 1.25 | 1.00 |
| Right atrium body size (mm) | 1.75 | 1.50 | 1.50 | 1.50 |
| Cannula body size (mm) | 1.25 | 1.25 | 0.75 | 0.50 |
